# Lecture 3—Measuring Pharmacy’s Work in the 21st Century

**DOI:** 10.3390/pharmacy6030063

**Published:** 2018-07-04

**Authors:** Richard H. Parrish

**Affiliations:** 1 St. Christopher’s Hospital for Children, Department of Pharmacy Services, 160 East Erie Avenue, Philadelphia, PA 19134, USA; richardhenryparrish2@gmail.com; Tel.: +1-215-427-5317; 2 School of Pharmacy, Virginia Commonwealth University, Richmond, VA 23298, USA

**Keywords:** key performance indicators, mission, vision, values, “hobby-pharming”

## Abstract

The importance of developing a thoroughly shared understanding of mission, vision, and values is highlighted in reference to the creation of meaningful and sustainable key performance indicators (KPIs). A review of clinical practice KPIs (cpKPIs) and operational KPIs (opKPIs) is provided using work load measurement activities from Canada, its province of Alberta, and the United Kingdom. In order for Singaporean pharmacy clinicians and leaders to embrace a unified KPI system, the natural tendency to measure what is easy and available, instead of what matters to patients, is difficult but must be overcome.

## 1. Introduction

Ask 10 pharmacists how to capture their workload on any aspect of the practice, and you will get at least 25 good answers. The problem with workload measurement is that the work of the pharmacist and pharmacy technician are changing rapidly. There are four huge temptations to overcome when deciding what to count as work: (1) the rush to fix workload measurement without a thoroughly shared understanding of mission, vision, and values; (2) output measurement in terms of either revenue generation or of cost avoidance (the private good versus public utility argument); (3) casual empiricism, i.e., the sole reliance on anecdotes, stories, and experiences to base one’s actions; and (4) the unchecked proliferation of so-called “hobby-pharming,” with a ‘ph’ for the ‘f’ in pharming. As mentioned in the prior lectures on clinical practice and formularies, and with Ken Barker’s admonition about pseudopharmacy in mind, let’s dig a bit deeper into these tendencies in order to discover the true nature of workload measurement as well as set up the next two lectures on the application of dashboards and the development of predictive analytics for managing those pharmacist and technician activities that really matter to patients. Only when we think and act in terms of what the patient needs from us will we be able to see through their eyes, act responsively, and generate healing. We are, after all is said and done, in a healing profession. And, it also has been said, “If we always do what we always did, we will always get what we always got.”

## 2. Deriving a Thoroughly Shared Understanding of Mission, Vision, and Values

How many of you have ever written a Mission, Vision, and Values (MVV) statement for your organization or your own practice? If you have, what was your experience like? If you haven’t, I would encourage you to be honest with yourself about what and how you have accomplished anything. One of my mentors, Professor Don Rucker, helped to mold Medicare in the early 1960s. He used to say that many think they get things done by dumping the problem into the barrel of inaction: hope, pray, finesse, and curse. I’ve found myself doing this on occasion. And, what got me out of the barrel was just thinking about how much more I could achieve when my actions were derived from a clearly articulated mission. The essential questions to ask in this regard are: why am I a pharmacist? What gets me out of bed every day, excited about my work? How do I relate to others? If you can answer these questions succinctly, you are on your way to a goal-directed mission of your practice.

The next element is vision. Vision has been described in terms of looking at what everyone looks at, and seeing what no know else sees. Vision is a long-term view of where you are going in terms of strategy and tactics. If you cannot see where you are at and where you are going, any destination will do. I do not mean to imply that having vision is equated with clairvoyance or omniscience. I believe that these parlor tricks are nothing more than “smoke and mirrors,” something to amuse you at the circus. But, not when we are talking about practice visioning. And, the bigger the vision, the better, through a goal process that some have called “BHAG,” Big Hairy Audacious Goals. According to Collins and Porras, a BHAG is a long-term goal that fundamentally changes the nature of a business [1].

BHAG comes from visionary “what-if” statements. What-if clinical pharmacists practiced in collaboration with other healthcare providers because this structure was found to be the most beneficial for patients? What-if the work of the clinical pharmacist was to identify and mitigate drug therapy problems? What-if clinical pharmacists realized just how vital is their patient care function in Singapore? What-if technicians were allowed to receive and check prescriptions, gather clinical data, and draw blood for point-of-care testing? The National Association of Governors (NAG) in the United States understands and articulated very clearly the clinical pharmacy mission and its impact on residents of their respective states. In a 2015 paper called “The Expanding Role of Pharmacists in a Transformed Health Care System”, NAG concluded that:

“The integration of pharmacists into team-based models of care could potentially lead to improved health outcomes. To realize that prospect, states should consider engaging in coordinated efforts to address the greatest challenges pharmacists face: restrictions in [collaborative practice agreements] CPAs, recognition of pharmacists as health care providers to ensure compensation for direct patient care services, and access to [health information technology] IT systems. Examining state-specific challenges and promising practices from other states will allow states to develop policies that permit pharmacists to practice within the full scope of their professional training across the health care continuum”.

[2]

Could there be any clearer definition of individual and societal need?

The third component of MVV is values. It has been said that we all live our lives in the image of our values, and attempt to re-create the world every day from this premise. What are the values that guide your practice? Which ones do you share with other providers? The standards of practice for Singapore, like other jurisdictions, express a number of professional values upon which the clinical practice of pharmacy is founded. In Alberta [3], these ethical standards are clear-cut, derived, and shared among pharmacists on the clinical register:

“Pharmacists and pharmacy technicians use their knowledge, skills and resources to serve patients, contribute to society, and act as stewards of their professions.

PATIENTS

Principle 1: Hold the well-being of each patient to be my primary consideration

Principle 2: Respect each patient’s autonomy and dignity

Principle 3: Maintain a professional relationship with each patient

Principle 4: Respect each patient’s right to confidentiality

Principle 5: Respect each patient’s right to healthcare

SOCIETY

Principle 6: Advance public health and prevent disease

Principle 7: Use health resources responsibly

Principle 8: Serve as an essential health resource

PROFESSION

Principle 9: Ensure that I am competent

Principle 10: Act with honesty and integrity

Principle 11: Demonstrate responsibility for self and other health professionals

Principle 12: Nurture the profession”

These ethical principles guide all decision making in Alberta regarding professional functions and conduct as well as the adjudication of misconduct and practice excellence, and are very similar to those in Singapore and in the United Kingdom’s National Health Service (NHS). In reality, value is that which one acts to gain or keep, where Value equals Benefits minus Cost (and Cost includes economic risk) [4]. The process both in Alberta and in the United Kingdom has been very intentional and focused on professional standards for developing a relationship with every patient in one’s care, and collaborating with the healthcare team to identify, prevent, and resolve drug therapy problems.

## 3. The Problems with “Clinical Interventions” and Current Key Performance Indicator (KPI) Measurement Efforts

When it comes to output measurement, clinical pharmacy historically has collected data based on an ill-defined construct called, “clinical intervention.” As discussed in the first lecture, an intervention in clinical pharmacy purports to be what pharmacists do in response to the errors of omission or commission of other healthcare providers. This construct describes value from the pharmacist’s perspective, and data is captured in terms of whether the physician accepted the recommendation subsumed in the intervention. If the recommendation was accepted, we gave ourselves a big pat on the back, and went on to find more interventions to make. However illusionary, this one statistic gave us the courage to soldier-on. Often missing in these interventions is output or outcome measurement in terms of either revenue generation (green dollar) or cost avoidance (red dollar), the so-called private good vs. public utility proposition. In and of itself, calculating economic value derived from either model does not tell the entire story of clinical pharmacy success. However, that fact doesn’t seem to faze our colleagues who predicate clinical pharmacy’s value on cost containment. How patients benefit is usually left out of their equations because it’s really difficult to meter out clinical pharmacy’s contribution to any patient’s outcome. The difficulty arises because often there is no linking of indication or reason for use to output and outcomes databases.

Our tendency to overgeneralize based on casual empiricism, or the sole reliance on anecdotes, stories, and experiences (or appearances) to base our actions, can misdirect efforts for rational explanation. The economist Milton Freidman was clear on how appearances can be misleading; he states it behooves us to test implications rather than assumptions in any sphere of economic and social life [5]. Applied to clinical pharmacy workload measurement, many gurus in the field posit the “low hanging fruit” theory as the basis for accurate indicators. Such approaches fall short of meaningful and sustainable practice activity measurement. Indeed, appearance management may be popular in some circles; however, what is needed is precisely a value proposition from the patient’s perspective. In Canada over the last several years, efforts to derive clinical key performance indicators or cpKPIs have led to the creation of a consensus statement and a charge to apply this intelligence through knowledge mobilization [6]. These efforts, coupled with changes in practice acts in the provinces, have produced a certain consistency about purpose, functions, and impact on outcomes for patients. For example, medication reconciliation on admission and at discharge, inter-professional rounds, and patient education during hospital stay and at discharge are included with pharmaceutical care plan, drug therapy problems, and “bundled” patient care “interventions.” Some of these cpKPIs are tasks (medication reconciliation and patient education), some are assessment (drug therapy problems), and some are planning (pharmaceutical care plan). Derived from a modified Delphi process, each cpKPI has an operational definition that guides clinical practice, data collection, and reporting. The Canadian Society of Hospital Pharmacists identified eight important caveats in the cpKPI process that includes:

(1) cpKPIs pertain to inpatients only;
(2) the number of admissions is the denominator for calculating cpKPIs;
(3) complexity does not affect what is measured;
(4) continual measurement is suggested;
(5) documentation of cpKPI is recommended highly;
(6) the pharmaceutical care plan differs from the drug therapy problems cpKPI;
(7) different pharmacists may be involved during provision of bundled patient care; and
(8) consensus was not reached for drug- and disease-specific quality indicators [7].

While this document represents one of the most organized and sustained efforts to quantify clinical workload ever attempted, it does not take into account several important factors in overall practice performance. First, cpKPIs pertain to inpatients, not hospital outpatients, emergency department patients, or ambulatory care patients seen in the medical office. Second, acuity, intensity, or complexity is not accounted for in any measurements. In fact, we have no empirically tested intensity measurement in clinical pharmacy. However, we will discuss one approach based on the number of medical conditions, the number of drug therapy problems identified or managed, and the amount of time spent with each patient. Third, the notion of the “bundled intervention” merits additional dialogue before wholesale adoption. This term has never been the object of any systematic study devoted to clinical pharmacy work or workload measurement. Finally, there is no agreement on drug- or disease-specific quality indicators. Each of these limitations represents an opportunity for Singapore pharmacy and health leaders to apply a magnifying glass to identify and determine the value proposition for clinical pharmacy vis-à-vis population health and predictive analytics. 

Let’s take the emergency department (ED) patient as an example of how to “blow out” cpKPIs for these patients. There are three types of patients that present to EDs: (1) treat and release; (2) treat and observe for a limited amount of time; and (3) treat and admit. Preliminary unpublished data collected from the ED of one of the largest tertiary care hospitals in Alberta Health Services identified a dichotomy of clinical pharmacist involvement in direct care for the function of medication reconciliation and rounding on teams. In this hospital, there was no pharmacist assigned to the ED. On the one hand, pharmacists attached to teams saw their team’s patients in the ED in almost 75% of cases admitted to hospital. Conversely, non-pharmacists performed medication reconciliation in the other 25% of cases; pharmacists did not see the patient until admitted. What about the “treat and release” and “treat and observe” sub-populations? These represent unexplored areas for consideration in Singaporean hospitals and urgent care centers. Moreover, both the complexity and specificity of diseases and comorbidities drives pharmacist direct care, and has to be incorporated into any comprehensive measurement system.

The last point on the tendency to develop KPIs that measure only what matters to pharmacy is the unchecked proliferation of what one of my former supervisors called, “hobby-pharming.” “Hobby pharming” is derived from hobby farming, an avocation that entails “doing what is within my comfort zone” or “doing what I like or have done before.” According to one popular definitional website, pharming is defined as “the production of pharmaceuticals from genetically altered plants or animals” [8]. However, this is not what I am talking about. “Hobby pharming” is a tongue in cheek phrase that describes a sort of “niche” or “micro” approach to professional practice. For example, providing a pharmacokinetic consultation for phenytoin, but not the pharmacokinetic (PK) assessment and plan for any of the patient’s other drug therapy problems. “I only do this, not that.”

Further to these points, cpKPIs and operational KPIs (opKPIs) need to dovetail with each other to form a comprehensive snapshot of activity and impact in real-time. The opKPIs refer to the aspects of practice devoted to drug preparation and distribution. I have said time and again that if the right medicine is not where it needs to be, at the right time, in the right dose and dose form, free of producing interactions or allergies, cpKPIs don’t really matter. If the operation cannot meet its service objectives, all the clinical pharmacy brought to the bedside will be for naught. Credibility dwindles when both aspects of the practice are not in sync. Operationally speaking, a number of experts recommend the use of the following indicators: (1) doses dispensed (multiplied by the volume for oral liquid and intravenous doses) or administered; (2) the number of orders verified, including outpatient chemotherapy; (3) inventory turnover; (4) human resource utilization and turnover; (5) percent of overtime; and (6) cost per patient for high cost drugs, high burden diseases, antimicrobials, and disease-modifying agents. Each of these parameters provides vital information about the operation’s efficiency and quality, and they can be integrated into cpKPIs to determine their optimal relationships, much like the purpose that financial ratios serve for managing expenses, liquidity, and profit.

## 4. Closing Remarks

Overcoming our natural tendency to measure what is easy and available, rather than what matters to patients, is often difficult. Change requires that each person embrace new definitions of work life and measurement. Identifying the drivers of your practices at each site will depend on the depth you are willing to go as well as the transparency of inquiry and contribution. I can tell you that from my experience in practice in nine US jurisdictions, one Canadian province, and an academic practice in New Zealand, a MVV approach that sets aside your biases and history will be successful. After changing a hospital pharmacy department into a pharmaceutical care practice over 25 years ago, on a cognitive level, many of my pharmacists embraced the concept of pharmaceutical care as a best practice methodology to propel the profession and their practices. Moreover, while outside consultant companies and services give ample direction on endpoints, few actually have worked through the processes needed to arrive at the intended destination. Even fewer understand the profound historical transformation taking place in the spectrum of care in pharmacy in the detail necessary to re-invent practice from its elements. In our next roundtable, we will work through an exercise and do the heavy lifting of change that I call “Job Parts” to facilitate change in direction from a departmental to a clinical practice or patient-focused model.
